# Unsedated peroral wireless pH capsule placement vs. standard pH testing: A randomized study and cost analysis

**DOI:** 10.1186/1471-230X-12-58

**Published:** 2012-05-31

**Authors:** Christopher N Andrews, Daniel C Sadowski, Adriana Lazarescu, Chad Williams, Emil Neshev, Martin Storr, Flora Au, Steven J Heitman

**Affiliations:** 1 Division of Gastroenterology, University of Calgary, Calgary, Canada; 2 GI Motility Lab, University of Alberta Hospital, Edmonton, AB, Canada; 3 Ludwig Maximilians University, Munich, Germany

**Keywords:** Esophagus, Gastroesophageal reflux disease, pH-metry, Clinical trial

## Abstract

**Background:**

Wireless capsule pH-metry (WC) is better tolerated than standard nasal pH catheter (SC), but endoscopic placement is expensive. Aims: to confirm that non-endoscopic peroral manometric placement of WC is as effective and better tolerated than SC and to perform a cost analysis of the available esophageal pH-metry methods.

**Methods:**

Randomized trial at 2 centers. Patients referred for esophageal pH testing were randomly assigned to WC with unsedated peroral placement or SC after esophageal manometry (ESM). Primary outcome was overall discomfort with pH-metry. Costs of 3 different pH-metry strategies were analyzed: 1) ESM + SC, 2) ESM + WC and 3) endoscopically placed WC (EGD + WC) using publicly funded health care system perspective.

**Results:**

86 patients (mean age 51 ± 2 years, 71% female) were enrolled. Overall discomfort score was less in WC than in SC patients (26 ± 4 mm vs 39 ± 4 mm VAS, respectively, p = 0.012) but there were no significant group differences in throat, chest, or overall discomfort during placement. Overall failure rate was 7% in the SC group vs 12% in the WC group (p = 0.71). Per patient costs ($Canadian) were $1475 for EGD + WC, $1014 for ESM + WC, and $906 for ESM + SC. Decreasing the failure rate of ESM + WC from 12% to 5% decreased the cost of ESM + WC to $991. The ESM + SC and ESM + WC strategies became equivalent when the cost of the WC device was dropped from $292 to $193.

**Conclusions:**

Unsedated peroral WC insertion is better tolerated than SC pH-metry both overall and during placement. Although WC is more costly, the extra expense is partially offset when the higher patient and caregiver time costs of SC are considered.

**Trial registration:**

Clinicaltrials.gov Identifier NCT01364610

## Background

Gastroesophageal reflux disease (GERD) is a common disorder defined as mucosal damage or typical symptoms produced by the abnormal reflux of gastric contents into the esophagus [1]. Ambulatory esophageal pH monitoring (pH-metry) is the established standard method for the diagnosis of GERD. Two methods are commercially available for esophageal pH-metry: standard catheter (SC) and wireless capsule (WC).

Standard catheter pH-metry involves the placement of a thin tube with one or more pH probes through the patient’s nose to 5 cm above the lower esophageal sphincter (LES), with the external end connected to a data recorder worn on the body [1]. The catheter is then left in situ for 24 hours. Although SC remains the most commonly used method for evaluating GERD, it has significant limitations. Patients often experience discomfort and embarrassment during SC testing leading them to alter their usual daily activities including their normal eating, drinking, and sleeping patterns. Patients also frequently decrease their physical activity. These lifestyle changes may reduce reflux events leading to test results that underestimate disease severity [1,2]. Finally, although SC is relatively inexpensive, most patients feel they cannot work during the testing period increasing the costs borne by patients [3].

The WC method (Bravo™ System, Given Imaging, Yoqneam, Israel; formerly produced by Medtronic Inc., Minneapolis, USA) uses wireless telemetry technology eliminating the need for a transnasal catheter. The small capsule with an embedded pH probe is affixed to the distal esophageal mucosa which conveys signals to a receiver/recorder worn on the body [4]. Wireless capsule placement can be performed endoscopically or manometrically and data can be collected for 48 hours or longer.

Wireless capsule has been shown to be safe [1] and as sensitive as SC for acid detection [5-7]. Furthermore, the lack of a transnasal catheter permits patient to continue their usual daily activities without impediment. However, WC also has some disadvantages. The sedation used during the standard endoscopic placement technique may affect the accuracy of the data collected during the first 24 hours [8,9]. Endoscopy also increases patient risk (due to the low but measurable risk of perforation, bleeding, or adverse reactions to sedation used) and increases the cost of the procedure. Consequently, other groups have evaluated the potential for trans-oral placement using manometry to locate the LES. A technical problem with this approach is that the esophageal manometry catheter is typically placed transnasally making precise location of the LES via a transoral approach potentially inaccurate. Lacy and colleagues validated a correction factor of 4 cm allowing for accurate placement of a transoral capsule [10]. They also determined that trans-oral placement was effective and well tolerated by patients. However, this was a non-randomized study and thus confounding by assignment bias may have overestimated the tolerability of manometric placement. Finally, although a cost analysis on endoscopically-placed WC has been performed [11], no study has assessed this question with transoral placement or from the perspective of a publicly funded health care system where time costs to patients and caregivers should be considered [12].

Our study had two aims. The first was to confirm that non-endoscopic peroral manometric placement of WC is as effective and better tolerated than SC using a randomized study design. Secondly, we performed a cost analysis of the available ambulatory esophageal pH monitoring techniques, including endoscopic WC placement (which was not assessed in this clinical trial). The data from the clinical study and the published literature were used to inform the cost analysis.

## Methods

### Study design

This was a dual center, randomized, non-blinded study. Patients referred for pH-metry for investigation of persistent symptoms possibly related to GERD were randomly assigned to standard trans-nasal catheter (SC) or wireless capsule (WC) pH testing using unsedated peroral placement. The study was performed at two regional motility centers in Alberta, Canada (Rockyview General Hospital, Calgary and University of Alberta Hospital, Edmonton). Participants in the WC arm were studied for either 24 hours (Calgary) or 48 hours (Edmonton). This approach was used so that a directly comparable timeframe of WC to standard SC testing (i.e. 24 hours) as well as usual WC practice (i.e. 48 hours) to standard SC practice were studied. All patients had traditional esophageal manometry to determine the distance to the LES. The study protocol was approved by the Health Research Ethics Boards of the Universities of Calgary and Alberta (registered at clinicaltrials.gov NCT01364610). Written and oral informed consent was obtained from all participants before they entered the study. The trial was performed in accordance with the International Conference on Harmonization of Good Clinical Practice guidelines, which have their origins in the Declaration of Helsinki.

### Patient population

Patients aged 18–75 years referred for ambulatory pH testing between August 2008 and August 2009 were approached for enrollment into the study. Patients with previous esophageal surgery (e.g. fundoplication, myotomy) or achalasia were excluded. Once enrolled, patients were randomly assigned to SC or WC. Randomization schedules were generated in advance in random blocks of 2 or 4, separately for each site (PROC PLAN, SAS 9.2, Cary, NC) and in separate envelopes. No stratification was used. Assignments were not blinded, but patients and investigators were not informed of their assignment until after the manometry on the day of the test. Patients who declined the study were provided standard care with SC pH-metry. Patients were not remunerated for participating. No enrolled patients dropped out or withdrew their consent. Recruitment was stopped after both sites had completed 20 WC and 20 SC studies.

The primary endpoint was the overall discomfort reported during WC versus SC pH-metry. Secondary endpoints included the success rates of probe placement, the site-specific discomfort of the manometry and pH probe placement procedures, the ability to perform usual activities, and the time off work between groups. Failure was defined as the inability to obtain a minimum of 20 hours of pH recording, and was subdivided into technical causes (e.g. equipment malfunction), and patient causes (e.g. inability to tolerate the insertion or ambulatory component of the pH probe).

### Esophageal manometry

Transnasal esophageal manometry (ESM) was performed in all patients according to standard procedures [13,14], with characterization of LES and esophageal function. Topical nasal anesthesia with viscous lidocaine was used.

### pH-metry

Standard Catheter (SC). After manometry, a calibrated standard antimony-based pH catheter (Comfortec Plus catheter and ZepHr Sleuth recorder, Sandhill Scientific, Inc., Highlands Ranch, CO) was inserted trans-nasally to 5 cm above the proximal border of the LES, affixed to the nose, and connected to the data recorder.

Wireless Capsule (WC). After topical pharyngeal anesthesia, the activated and calibrated WC (Bravo wireless pH capsule system, Medtronic Inc., Minneapolis MN) was inserted by mouth using the single-use Delivery System to 5 cm above the proximal border of the LES. To convert the distance from the naris (obtained at manometry) to the distance from the incisors for peroral WC placement, a conversion factor of 4 cm was subtracted [10]. WC was deployed as per the manufacturer’s instructions. Briefly, suction was applied to the delivery system at a stable pressure of approximately 500 mmHg for 30-60 seconds to bring the esophageal mucosa into the suction well, then a spring-loaded pin was deployed within the suction well to affix the capsule. The delivery catheter was then removed.

After probe placement in both cases, responsiveness to weakly acidic liquids (apple juice) was ascertained on the data recorder in real time. Specific symptom events were recorded by pushing buttons on the recorder and using a written diary. After the study, the SC was removed. The WC detaches spontaneously, typically after 5–7 days, and passes out in the stool. The pH study results were evaluated using standard composite scores provided with the respective analysis software.

### Questionnaires

Each patient completed a short symptom questionnaire following pH probe placement and a second short questionnaire 24–48 hours later when they returned to complete the test. Patients in the 24 hour study in Calgary were additionally contacted after the study to assess the effect of the test on their work patterns. Assessment of discomfort levels was done using a 10 cm visual analog scale (VAS) [15] after the manometry, after the device placement and after the pH study period was completed.

### Sample size and statistical analysis

The study was independently powered at both sites (>80%) to show a significant difference in the primary outcome measure with a minimum 20 patients per group based on overall discomfort on a 10 cm VAS scale similar to that done in a previous study [16]. The minimum clinically important difference for changes for patient-rated acute pain on this VAS scale is between 9 and 13 mm [17-19]. Normally distributed means were compared using Student *t*-test and count data were compared using Pearson Chi-square with continuity correction, or Fisher Exact test where cell counts were 5 or less. A p-value < 0.05 was used to assess significance. All tests were two-tailed.

### Cost analysis

We performed a cost analysis of 3 different strategies for obtaining esophageal pH-metry: 1) ESM + SC, 2) ESM guided WC (ESM + WC) and 3) endoscopically placed WC (EGD + WC). A decision tree was constructed using decision analysis software (TreeAge Pro Suite 2009, Williamstown, MA, USA) and is depicted in Additional file 1: Figure S1. The perspective of the base case analysis was that of the publicly funded health care system. Given the short time horizon of the analysis (24 hours), no discounting was performed.

### Model inputs

The initial success rates for ESM + WC and ESM + SC based on our trial results were 88% and 93%, respectively. A 95% initial success rate for EGD + WC was assumed from published studies in the recent literature, which range from 90 to 100 percent [3,5,10,20,21]. In the EGD + WC strategy we assumed that a second WC would be successful in those where the first attempt failed. In this study a second attempt was successful in both the ESM + WC and ESM + SC strategies for those willing to undergo a repeat procedure (80% and 33%, respectively). Further detail regarding the placement and insertion failures are found below.

In the base case analysis, costs were those relevant to a publicly funded health care system and thus included patient and caregiver time costs in keeping with Canadian guidelines [12]. Also consistent with contemporary guidelines and the perspective of the publicly funded health care system, costs resulting from lost productivity were not considered. Thus costs resulting from reduced work capacity due to illness and costs borne by employers to hire and train replacement workers for patients during absences were not included. Based on our study findings, we assumed that 100% of patients in the ESM + SC strategy would choose not to work in contrast to the ESM + WC strategy where 93% of patients would have worked during the testing period (see Results). However, we assumed that patients would need 2 hours off work for the procedure in the ESM + WC strategy. For those in the EGD + WC strategy we assumed that patients would not return to work until the next day given the sedation. Furthermore, it was assumed that a companion would be required for a total of 4 hours to drive the patient to and from the appointment [22]. We valued each hour of patient and caregiver time at the average hourly wage rate ($22.50 per hour) published for Albertans in 2010 as was done in a previous study [22].

The direct costs of each of the procedures included the non-physician costs (capital, nursing, drugs, cleaning costs, etc.) which we obtained from the Alberta Health Services - Calgary Zone costing database [23] and the physician consultant and procedural fees for the procedures. The cost of WC was provided by the manufacturer. All costs are reported in 2010 Canadian dollars. A summary of the input values used in the decision tree is shown in Table 1.

**Table 1:** Cost Analysis: Base-case Input Variables

**Failure rates (1st attempt)**	**Value (%)**
EGD + WC	5
ESM + WC	12
ESM + SC	7
**Failure rates (2**^**nd**^attempt)*	
EGD + WC	0%
ESM + WC	20%
ESM + SC	67%
**Costs (Direct)**	**Value ($ CAN)**
EGD non-physician	587.95
EGD physician	124.86
ESM non-physician	408.56
ESM physician	103.22
WC non-physician	292.29
WC physician	125.62
SC non-physician	87.89
SC physician	125.62
**Costs (Indirect)**	
EGD + WC	315.00
ESM + WC	45.00
ESM + SC	180.00

EGD, esophagoduodenoscopy; ESM, esophageal manometry; SC, standard pH catheter; WC, wireless pH capsule.

* 2^nd^ attempt failures due to patients not wanting the test repeated.

Total cost EGD + WC = $1,445.72

Total cost ESM + WC = $974.69

Total cost ESM + SC = $905.29

Sensitivity analyses were performed. In addition we examined a scenario where patient and caregiver time costs were excluded.

## Results

A total of 86 patients had esophageal manometry and were enrolled in the study (46 in Calgary and 40 in Edmonton). There were no significant differences in the baseline characteristics or variables tested between the two study sites (data not shown) and thus the data presented in the following tables are the pooled results for both sites. There were no significant differences between the SC and WC groups in age, indication for testing, or manometry results (Table 2). Although there was a trend toward more females in the WC group, this did not reach statistical significance.

**Table 2:** Patient demographics

**Variable**	**SC Group**	**WC Group**	**P value**
	**n = 43**	**n = 43**	
Female (%)	26 (60)	35 (81)	0.057
Age (mean years ± SE)	49 ± 2	52 ± 2	0.328
Indication for Testing (%)			0.831
Reflux Symptoms	30 (70)	33 (77)	
Chest Pain	8 (19)	7 (16)	
Dysphagia	4 (9)	2 (5)	
Other	1 (2)	1 (2)	
Total Procedure Duration (manometry	53 ± 4	62 ± 8	0.339
and pH probe insertion, minutes ± SE)			
**Manometry Result** (%)			0.377
Normal	12 (29)	17 (44)	
Low LES resting pressure	18 (43)	12 (31)	
IEM	7 (17)	8 (21)	
Spastic (DES/nutcracker)	3 (7)	2(5)	
Other	2 (5)	0 (0)	
**pH Results at Completion**			
Elevated Esophageal Acid Exposure*	24 (56)	19 (44)	0.388

*Based on standard composite scores. Cutoff for SC: Johnston/DeMeester, normal score <22. Cutoff for WC: DeMeester, normal score <14.7. DES, diffuse esophageal spasm; IEM, ineffectual esophageal motility; LES, lower esophageal sphincter; SE, standard error; SC, standard pH catheter; WC, wireless pH capsule.

### Manometry and pH probe placement

The manometric assessment was well tolerated by both SC and WC groups, with no significant differences in overall or site-specific discomfort (Table 3). The placement of the SC probe was more uncomfortable in the nasal region compared to WC (36 ± 4 mm in the SC group compared to 6 ± 2 mm in WC, p < 0.001), but there were no significant differences in throat, chest, or overall discomfort between the groups during placement. The time required for the combined manometry and pH probe placement was not significantly different between groups (Table 2).

**Table 3:** Grouped Outcomes

**Variable**	**SC Group**	**WC Group**	**P value**
	**n = 43**	**n = 43**	
**Manometry Discomfort**^a^			
(mm VAS ± SE)			
Nasal	40 ± 3	33 ± 4	0.320
Throat	40 ± 3	34 ± 3	0.165
Chest	15 ± 3	14 ± 3	0.738
Overall	39 ± 3	34 ± 3	0.285
**pH Placement Discomfort**^a^			
(mm VAS ± SE)			
Nasal	36 ± 4	6 ± 2	<0.001
Throat	37 ± 3	32 ± 4	0.317
Chest	13 ± 3	14 ± 3	0.968
Overall	33 ± 3	29 ± 4	0.406
**pH Test Discomfort**^a^			
(mm VAS ± SE)			
Nasal	39 ± 3	10 ± 3	<0.001
Throat	43 ± 4	19 ± 4	<0.001
Chest	14 ± 3	29 ± 4	0.001
Overall	39 ± 4	26 ± 4	0.012
Eating and Drinking (mm VAS ± SE,	51 ± 4	75 ± 5	<0.001
100 = completely normal)			
Ability to do Usual Activities (mm	75 ± 5	92 ± 2	<0.001
VAS ± SE, 100 = completely normal)			
Would you Repeat the Test Again?	51	88	<0.001
(% Yes)			

^a^100 = worst discomfort. SE, standard error; SC, standard pH catheter; WC, wireless pH capsule; VAS, visual analogue scale.

### pH study discomfort

At the completion of the pH assessment, the overall discomfort score was less in the WC group than the SC group (26 ± 4 mm vs 39 ± 4 mm, respectively, p = 0.012; Table 3). The nasal and throat discomfort scores were lower, but the chest discomfort scores were higher in the WC group compared to the SC group (Table 3). Patients in the WC group were better able to eat and drink without difficulty and also reported being more likely to undertake their regular daily activities (p < 0.001 for both). Finally, those in the WC group reported a higher willingness to undergo the pH procedure again (88% vs 51%, p < 0.001). The proportion of pH studies showing increased acid exposure was not significantly different between the groups (Table 2).

Very few patients attempted to perform their usual work activities during the pH study (data not shown). Since subjects were not aware of their assignment until the day of the procedure, most planned in advance to take the day off work. However, when surveyed after the pH study none of the patients in the SC group felt they could have performed their regular work duties. In contrast, 93% of the patients in the WC group reported that they would have continued their usual daily routine including going to work (p < 0.0001).

### Failure rates

Three of 43 (7%) studies were unsuccessful in the SC group (1 equipment malfunction after the patient dropped the recorder and 2 patients were unable to tolerate the indwelling nasal pH catheter with subsequent early probe removal). In the WC group, 5 of 43 (12%) of studies were unsuccessful (2 capsules failed to calibrate, 2 capsules detached early, and one patient did not tolerate peroral insertion of the WC due to excessive gagging and anxiety) (Table 4). The 2 early detachments occurred within the first 5 WC studies that were performed. The overall failure rates were not different between the groups (p = 0.713).

**Table 4:** Initial Failures - Reasons and Rates

	**SC Group**	**WC Group**	**P**
	**n = 43**	**n = 43**	**value**
Total Failed (%)	3 (7)	5 (12)	0.713
Equipment Malfunction	1 (2)	2 (5)	
Immediate Detachment (<1 hour)		1 (2)	
Premature Detachment		1 (2)	
Could not tolerate peroral insertion		1 (2)	
Could not tolerate transnasal catheter	2 (5)		
(self-removal)			

### Cost analysis

The data reported in Table 5 represent the expected total cost to obtain 24-hour pH-metry per patient. Under the base case assumptions the endoscopically placed WC strategy was the most expensive strategy at $1475 per patient whereas the standard ESM + SC was the least costly at $906 per patient. The manometrically placed WC strategy (ESM + WC) was $1014 per patient or $108 more than ESM + SC (Table 5).

**Table 5:** Cost Analysis ($ CAN)

**Base-case**	**Cost**
EGD + WC	1474.80
ESM + WC	1014.40
ESM + SC	906.00
**Sensitivity analyses**	
ESM + WC with 2% failure	981.32
ESM + WC with 5% failure	991.26
ESM + WC with 15% failure	1024.39
ESM + SC with 90% absenteeism	887.97
**Threshold cost of WC**	192.91
**Time costs excluded**	
EGD + WC	1155.80
ESM + WC	968.60
ESM + SC	726.00

EGD, esophagoduodenoscopy; ESM, esophageal manometry; SC, standard pH catheter; WC, wireless pH capsule.

In a sensitivity analysis we decreased the failure rate of ESM + WC from 12% to 5% which decreased the cost of ESM + WC to $991 and further dropped it to $981 with a 2% failure rate (Table 5). If the work absenteeism in the ESM + SC group was decreased from 100% to 90% during the test, the cost of ESM + SC decreased to $888. In a threshold analysis using the base case assumptions, the SC and ESM + WC strategies became equivalent when the cost of the WC device was dropped from $292 to $193 (Table 5). When patient and caregiver time costs were excluded the expected costs of EGD + WC, ESM + WC and ESM + SC were $1,155.80, $968.60 and $726, respectively.

## Discussion

The results of this first randomized trial confirm that 24 hour pH-metry using unsedated peroral WC insertion is better tolerated than SC both overall and during placement. As expected, nasal and throat discomfort was prominent in the SC group, while chest discomfort was higher in the WC group during the pH monitoring period. Furthermore, in keeping with previous reports on WC, our findings suggest that the majority of patients undergoing WC would choose to continue their regular activities including employment, in contrast to SC where none reported being capable of working. Lastly, although WC is more costly than SC, our cost analysis shows that the extra expense of WC is partially offset when the higher patient and caregiver time costs of SC are considered.

Since SC and WC appear to be equally effective in diagnosis of abnormal reflux when the procedure is successful, failure rates thus become a key measure of effectiveness. The 12% overall failure rate of WC in the present study falls within the range of that reported in the literature [5,10,24], although a recent series reported a failure rate of only 2% [20]. The 5 WC failures that we encountered resulted from 2 capsule calibration errors (equipment failure), 1 patient who could not tolerate placement of the capsule (patient intolerance) and 2 early capsule detachments. Both of the WC early detachments occurred near the start of the trial. Although the investigators did have some prior experience with WC at the start of the study, it is certainly possible that the early detachments reflect the learning curve of the technique. However, it should also be noted that this study randomized patients prospectively which previous case series did not; since previous large series of WC did both endoscopic and unsedated transoral placements [10,21], patients who felt unable to tolerate unsedated placement presumably opted for endoscopic placement with sedation. That approach will underestimate failure rates from a patient intolerance standpoint, and thus the approach used in this study is more applicable to situations where endoscopic placement is not available. Although this study was not designed to evaluate the 4 cm nose-to-mouth conversion factor determined by Lacy et al. [10], there were no gross misplacements of WC judged either clinically or according to the pH study results using this method. Therefore, this relatively non-invasive technique is easy to learn and perform and is as reliable as SC for the ambulatory collection of esophageal pH data.

Placement of WC without need for direct visualization via gastroscopy is an important development. Endoscopy is resource intensive, carries risks to patients and the sedation that is generally required may alter the results during the first 24 hours of pH monitoring [9,10,25]. Furthermore, patients can’t drive or work after receiving sedation, which increases the patient-borne costs of the procedure. We have shown that this is also a significant problem for SC given that none of the patients in the SC group reported being capable of going to work while the catheter was in place. While there are some motivated patients in the community who choose to work with a SC in place, these are a small minority. In contrast, over 90% of those in the WC group would have chosen to return to work.

The results of our cost analysis demonstrate that peroral WC (ESM + WC) is more costly than ESM + SC, but less expensive than WC placed via gastroscopy (EGD + WC). However, the incremental cost of ESM + WC over ESM + SC drops when one takes into consideration the increased patient and caregiver time costs associated with SC. When time costs were excluded the incremental cost of ESM + WC over ESM + SC was $243 compared to $108 when these costs were included. Furthermore, had our failure rate been only 5% instead of 12%, the incremental cost of ESM + WC over ESM + SC would be $85 instead of $108. Our post procedure questionnaire suggests that patients prefer WC over SC given that nearly 90% would agree to have it repeated if necessary compared to only half of those who received SC. Therefore, given that patients prefer WC, can continue their usual daily activities and can continue to work during the ambulatory testing period, the added cost of WC may be something that health jurisdictions should consider. Like many technologies, the cost may drop over time. We have shown that WC would be cost neutral if the cost of the device were dropped to $193.

Our study has a number of limitations. Although the study was powered to assess the overall patient experience with pH placement and 24–48 hours of testing, the sample size may have been too small to detect significant differences in infrequent secondary outcomes such as failure rates. However, the failure rates did match those in the literature, and were subjected to sensitivity analysis in the cost analysis. Second, the VAS scales used to assess discomfort have not been validated specifically for manometry or pH testing, and thus may not accurately capture all dimensions of the pH testing experience. They are, however, generally accepted measures for pain [15], and have been used in the pH-metry literature previously [16]. With regard to the cost analysis, the costing model was based on a number of assumptions which may not be accurate for all populations or situations. However, this is a feature of all cost analyses. To account for the uncertainty in our model estimates we performed sensitivity analyses to test how changes in our model inputs affected our results. Finally, we assumed that SC and WC have equivalent effectiveness for diagnosis of GERD. Typically, WC studies are performed for 48 hours in clinical practice, and this extra data may improve sensitivity for detection of abnormal acid exposure [5]. However, there is no consensus as to whether this improves the effectiveness of the technology, but WC does not appear to be less sensitive than SC [5-7]. We thus took a conservative approach based on the assumption that the technologies were equivalent.

The major limitation of WC pH-metry is the pH measurement from only one site. SC typically has a proximal pH sensor as well, which provides more data, although does not affect composite score calculation and subsequent determination of abnormal acid exposure. With the advent of new technologies such as multichannel intraluminal impedance with pH (MII-pH), even more data is available to analyze all refluxate regardless of pH. This becomes increasingly important in the post-proton pump inhibitor (PPI) era, where the majority of pH investigations are done for atypical symptoms or PPI failure. The exact role of WC pH-metry in the stable of diagnostic tools for reflux disorders has yet to be fully determined, but will likely be the modality of choice for confirmation of acid reflux, for example prior to fundoplication.

## Conclusions

This randomized study adds to the growing body of literature that WC pH testing is clearly more acceptable to patients compared to SC-based methods. Until recently the increased cost, especially associated with endoscopy, has limited the uptake of WC in publically-funded health systems. However, unsedated peroral placement of WC is simple, effective, and as well tolerated as transnasal placement of a SC. Following WC placement, patients can resume their usual activities and return to work which is a significant advantage over SC. Lastly, although WC is more costly than SC, health jurisdictions should take into consideration that patient borne costs of WC are lower and that patients strongly prefer WC over SC.

## Disclosures

### Financial support

Medtronic Canada Inc. provided the Bravo equipment (hardware, vacuum apparatus, portable data recorders) free of charge but had no role in the study design, recruitment, administration, analysis of data, manuscript, or publication of the study. Bravo™ capsules were purchased by the investigators. Supported in part by the Noel Hershfield Fund for Training in Advanced Gastroenterology.

## Competing interests

The authors declare that they have no competing interests.

## Authors’ contributions

CNA was lead investigator, co-designed the study, provided the data analysis and co-wrote the manuscript. DCS was a co-investigator and co-wrote the manuscript. AL was a co-investigator and co-wrote the manuscript. CW was a co-investigator and provided administrative assistance. EN co-ordinated the study and questionnaires. MS was a co-investigator and co-wrote the manuscript. FA provided the cost data and assisted with the cost analysis. SJH co-designed the study, was co-investigator, performed the cost analysis and co-wrote the manuscript. Each author has approved the final draft submitted.

## Pre-publication history

The pre-publication history for this paper can be accessed here:

http://www.biomedcentral.com/1471-230X/12/58/prepub

## Supplementary Material

Additional file 1 **Figure S1.** Decision Tree for comparison of 3 pH testing strategies: endoscopic WC placement, manometric WC placement, and manometric SC placement. EGD, esophagogastroduodenoscopy; ESM, esophageal manometry; WC, wireless pH capsule; SC, standard pH catheter. Click here for file
