# Supplementary figures and images for: Unsedated peroral wireless pH capsule placement vs. standard pH testing: A randomized study and cost analysis
Source: BMC Gastroenterol. 2012 May 31;12:58. doi: 10.1186/1471-230X-12-58 (PMC3413593; doi:10.1186/1471-230X-12-58)

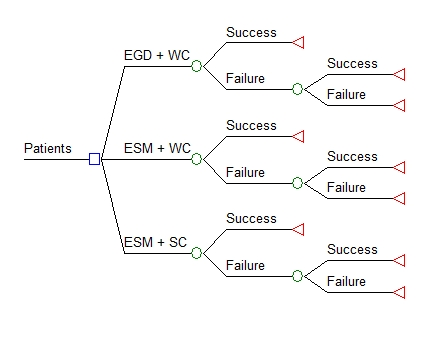

Supplement: Additional file 1 — Figure S1. Decision Tree for comparison of 3 pH testing strategies: endoscopic WC placement, manometric WC placement, and manometric SC placement. EGD, esophagogastroduodenoscopy; ESM, esophageal manometry; WC, wireless pH capsule; SC, standard pH catheter. [file 1471-230X-12-58-S1.tiff]
